# Wildlife as Food and Medicine in Brazil: A Neglected Zoonotic Risk?

**DOI:** 10.3390/pathogens13030222

**Published:** 2024-03-02

**Authors:** Caio Graco Zeppelini, Ianei de Oliveira Carneiro, Poliana Mascarenhas de Abreu, Ann Katelynn Linder, Romulo Romeu Nóbrega Alves, Federico Costa

**Affiliations:** 1 Instituto de Saúde Coletiva, Universidade Federal da Bahia, R. Basílio da Gama, s/n—Canela, Salvador 40110-040, Brazil; ianeica@gmail.com (I.d.O.C.); fcosta2001@gmail.com (F.C.); 2 Faculdade de Medicina Veterinária, Campus Tancredo Neves, Universidade Salvador (UNIFACS), Av. Tancredo Neves, 2131—Caminho das Árvores, Salvador 41820-021, Brazil; polimda@outlook.com; 3 Harvard Law School, 1585 Massachusetts Ave, Cambridge, MA 02138, USA; alinder@law.harvard.edu; 4 Programa de Pós-Graduação em Etnobiologia e Conservação da Natureza, Universidade Estadual da Paraíba, Rua Baraúnas, 351—Bairro Universitário, Campina Grande 58429-500, Brazil; romulo_nobrega@yahoo.com.br

**Keywords:** bushmeat, wet market, hunting, ethnobiology, zoonosis, zootherapy

## Abstract

The practice of consuming wild fauna in Brazil is both culturally and socioeconomically questionable. Wild animals and their byproducts are sought for nutritional, medicinal, and/or supernatural reasons, with some taxa (e.g., songbirds) being kept as pets. This practice is concentrated in traditional and rural communities, as well as the rural exodus populations in large urban centers, maintained both by cultural preferences and for their role in food safety in part of the rural exodus community. A total of 564 taxa are known to be sold in wet markets in Brazil, with birds, fish, and mammals being the most commonly listed. There is great zoonotic outbreak potential in this consumption chain given the diversity of species involved (with several listed being known reservoirs of zoonotic pathogens), invasion of wild environments for hunting, unsanitary processing of carcasses, and consumption of most/all biotopes of the animal, as well as the creation of favorable conditions to cross-species pathogen transmission. Given its socioeconomic situation and the global trends in disease emergence, there is a risk of the future emergence of a Public Health Emergency of International Concern in Brazil through wildlife consumption.

## 1. Introduction

Zoonotic pathogens are a matter of great concern to the public health community at large. The majority of novel human pathogens discovered in recent decades are of zoonotic origin [1,2], mainly from wild fauna, as a result of the continuous encroachment of sylvatic habitat by human activity [3]. Anthropic impacts on the biosphere and globalization have ushered in a new epidemiologic scenario, where diseases considered under control might reemerge (e.g., tularemia [4], Lassa fever [5], plague [6]), as well as give way to rising pathogens [7]. The latter was made clear with the pandemic spread of SARS-CoV-2, whose origin is likely animal, although this is not completely elucidated [8,9].

One of the main animal–human interfaces that allow a pathogen to jump from its original host into human populations is the consumption of wildlife. Wildlife is sought after in several contexts, such as a food source, human medicine (both physiological and metaphysical), veterinary medicine, and pet rearing [10,11,12,13,14].

Given their status as a potential point of contact with the transmission cycles of novel wild pathogens, wet markets (as a collective term for any sort of market or outlet that sells fresh foods and/or live animals) are places of high interest for One Health professionals, especially given the recent history of global health emergencies whose outbreaks can be traced back to fauna commercialized in them (e.g., avian influenza [15], SARS [16]); the ongoing COVID-19 pandemic is one of the more dire examples, with a current death toll of over 5.83 million people as of February 2022 [14,17].

Wet markets are a tradition in Brazil, presenting as open street fairs and public markets, occurring in almost every settlement or city. Brazilian wet markets present a wide offering of retail products, which include animals (domestic and wildlife) and animal byproducts [18,19,20,21]. Despite wildlife trafficking being a crime under Brazilian law, there is a growing ethnobiological record of this illegal commerce in wet markets (e.g., [11,22,23]), although the risks to public health in this human–wildlife interface remain mostly unexamined. Here, we characterize the situation of wildlife consumption in Brazil and discuss the potential for novel and reemerging zoonotic pathogen outbreaks arising from the chain of activities involved in this social phenomenon.

## 2. Methods

In order to characterize the practices of animal extractivism, consumption, and commerce, as well as contextualize it in terms of the risk of infectious disease transmission associated with the practice, between 15 and 20 March 2021, a nonexhaustive literature search was performed on Web of Science (https://app.webofknowledge.com/author/search (accessed on 15 March 2021)). The descriptors used in the search delimited the region of study (Brazil) and focused on the concepts of wild fauna and the activities in the wild fauna supply chain (e.g., “hunting”, “bushmeat”, “traditional remedy”, “folk medicine”). Entries focused on plants, and other nonanimal items were excluded. Data extraction focused on detecting information on (1) supply chains and commerce practices; (2) sourcing practices, techniques, and regions; (3) legal information regarding the practice, potential regulation, surveillance, and control measures; and (4) reports on zoonotic disease transmission involving bushmeat. Given the constant presence of inventory lists of species involved in the practices, a table was prepared to compile the available information on the taxa explored, their type of use (when discriminated), and what parts were used (Table S1). A table of the articles consulted for ethnobiological information and the type of study that originated the information is presented in Table S2. Of 39 publications with ethnobiological information consulted and maintained for the study, 31 contained data on the medicinal uses of wild animal products, while 12 contained information on their use as food.

## 3. Characterizing Wildlife Consumption in Brazil

### 3.1. Faunal Diversity Involved

Similar to Brazil’s high biodiversity, a large variety of taxa, both invertebrate (e.g., Annelida, Mollusca, Panarthropoda, Cnidaria) and vertebrate species, are consumed [10,13,24,25,26,27]. A nonexhaustive literature search retrieved records of 564 taxa being consumed in Brazilian territory (Table S1). Of the species with declared specific usage, most (230, 40.7%) were sought solely for medicinal purposes, 91 (16.1%) solely for food, and 203 (36%) for both. The taxonomic groups with the highest number of species and records found were mammals (85 species, 15%), birds (190, 33.6%), and fishes (102, 17.8%). Birds were also being traded as pets.

### 3.2. Uses and Culture

This phenomenon is part of a complex sociocultural network that can include traditional, indigenous, or rural communities and settlements [28], with heavier participation of the elder generations from lower socioeconomic status. There is a strong involvement of rural exodus and urban migrants from traditional communities [29,30,31,32].

**Figure 1:**
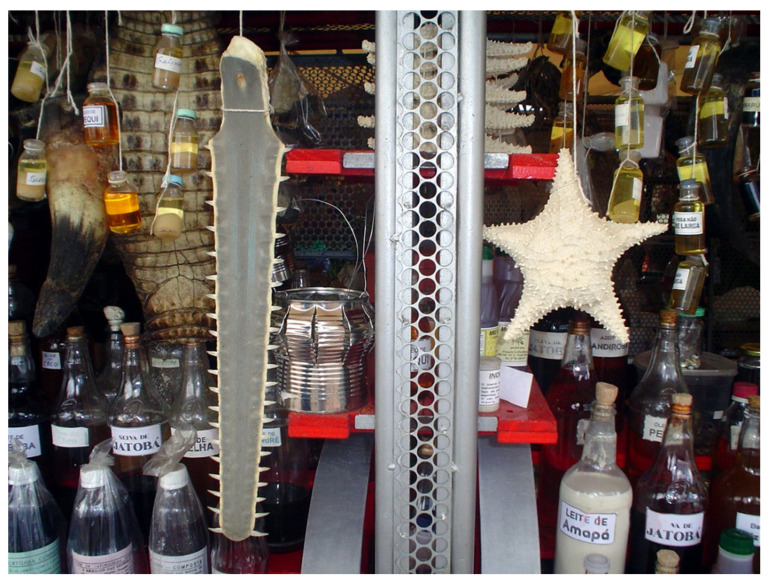
Zootherapic products exposed in urban vendors in Belém, State of Pará: yacare leather, rostral expansion on *Pristis perotteti*, and an exemplar of *Oreaster reticulatus*. Photo: Rômulo Alves.

Participation of bushmeat in the diet is likely linked to income, as extractivist activities can be a source of income and more affordable protein for lower-income families [12,33]. Animal-based medicine is divided between complementary alternative medicine (CAM) and its role as a substitute for allopathy in areas and contexts where access to a regular medical center is not viable [13,34]. The maintenance of supply chains is in part given by bushmeat as a source of accessible protein (by lower costs and by potentially being accessed directly by hunting) but also due to the cultural resilience of the ethnobiological and ethnopharmacological relationship of traditional communities and peoples with these resources [12,13,23,35]. The wares presented in wet markets can vary widely. Animals sold for food and medicine can be offered both alive and dead (some of the prescribed medical practices require a live animal), as a whole animal, as a treated carcass, or sold in pieces or processed (Figure 1) [26,33,36,37]. Medicines, both animal and herbal, are sold together in specialized vendors, with the vendor usually providing instructions for use.

### 3.3. Origins of Consumed Animals 

Most species traded are native to the area where they are sold [11,24,26,38,39]. However, certain taxa have high demand countrywide, with extractivist chains responsible for shipping and supplying the animal product over long distances, commonly in coast-to-continent routes or from the Amazon to the remaining territory [27,30,40,41].

### 3.4. Supply Chain and Associated Economic Activity

Fauna trafficking removes 38 million individuals from the wild and generates USD 2 billion [42]. The illegal status forces the supply chain to the underground, causing it to be poorly understood in spite of the increase in research interest in Brazil [11,13,39,43,44]. Particular taxa with ubiquitous high demands, such as *Podocnemis* turtles in the Amazon region, are better understood, given the size and accessibility of the operations [43]. The traffic of wild birds is the more understood of the supply chains, boasting profits estimated at USD 30/animal (at 2018 exchange rates), with rare taxa reaching USD 170 each [12]. Wild pets generate approximately USD 36 million BRL/year in the legal market alone [45], with increasing demand due to biophilia.

**Figure 2:**
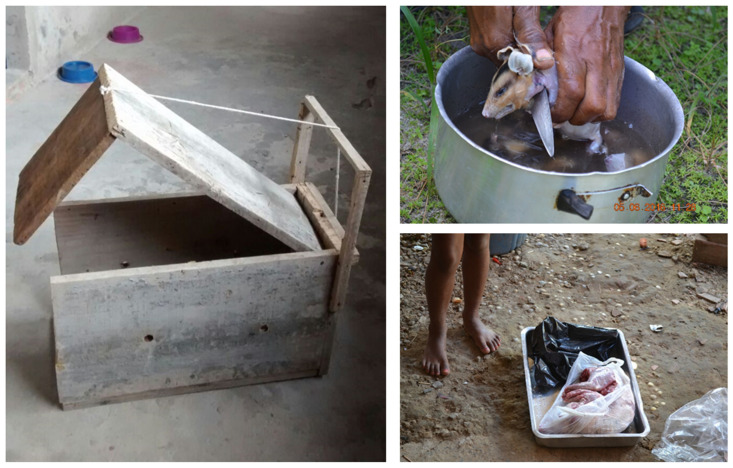
Different populations in Brazil have hunting as a source of animal protein. (**Left**) Trap made by hand for hunting small mammals; (**Right, above**) specimen of *Didelphis albiventris* being prepared by the hunter for consumption; and (**Right, below**) specimen of *Nasua nasua* packaged and without skin ready for consumption. Photo: Ianei Carneiro.

“Professional” hunting (i.e., people whose main source of income is hunting activities, in spite of its illegal status in Brazilian law) is commonly the job of locals, with middlemen funding part or all of the hunt with loans used for supplies and expenses [35]. Sourcing and preparation of specimens are carried out in mostly artisanal fashion with simple tools and employment of dog-aided hunting, traps, and firearms (Figure 2) [46,47,48]. 

Product transport can be the task of specialized smugglers or could involve professionals that work on long-distance travel (e.g., truck drivers, migrant rural workers) to avoid suspicion. Different products have their supply chains tailored to their particular characteristics. “Shelf-stable” products such as dry materials (teeth and bones, claws, horns, feathers, snake rattles, etc.), animal preserves (e.g., in alcohol or oil), tanned or sundried goods (leather, whole dry animals, etc.), and preparations (powdered products, concoctions) can be stored and sold at convenience. Perishable goods and other products that require refrigeration or freezing (meat, eggs, milk, viscera, blood, lard, suet, etc.) are normally obtained based on demand.

Exchange can take place in fairs and markets or directly in the client’s household [35]. Animals employed in Afro-Brazilian religious ceremonies can usually be sourced from stores that supply other types of products for this purpose [19], alongside other edible items, earthenware, musical instruments, and other utilities.

The relationship between public wet markets and wildlife trafficking is ubiquitous, and the target of regulation and law enforcement surveillance is to curb the practice [19]. As a response, less conspicuous ways have been procured to conduct business and avoid scrutiny. Alves and Rosa [18,49] observed organizational strategies, such as whole animals being stored in separate containers of bags, being exposed only during transactions, as a precaution to elude the environmental police.

Social media and instant messenger platforms such as Facebook and WhatsApp have gained popularity as marketplaces due to their accessibility, in many cases eliminating the need for middlemen [12,13]. Virtual environments have several important characteristics for this type of commerce, such as avoiding conspicuous gatherings, easy and wider access to vendors, and the gatekeeping measures made possible by digital means (e.g., closed groups with strict entry criteria and end-to-end encryption on messages that can be deleted from all devices involved simultaneously) [50].

## 4. Zoonotic Risk

Hunting is considered a high-risk zoonotic activity in Brazil, with hunter populations being considered potential sentinels for zoonotic spillover [30,51] and highly vulnerable to the acquisition of new and reemerging zoonotic pathogens [52,53]. Hunting is a risk exposure both directly, by the manipulation and treatment of the downed animals, and by moving the frontier of human activity into areas that favor pathogen transmission or into contact with novel transmission systems [52,54].

Animals are commonly consumed in their entirety, either as a single item or dismembered into different pieces/products [10]. This increases the amount of contact with the carcasses, the variety of tissues and organs with potentially distinct parasite groups (e.g., helminths and *Giardia* from the intestines, ectoparasites from the skin, or protist and bacterial pathogens from bodily fluids), and the amount and types of risk exposures in the process. Cross-contamination could occur in places where wild animal-based products and other foodstuffs are stored or transported together if inadequate hygiene and preservation conditions are not observed—something likely to happen in irregular activities such as bushmeat trafficking [29,35].

The absence of hygiene and biosafety standards on wild animal preparation and processing is another window for cross-contamination, as “cleaner” tissue (such as muscle or fat) could come into contact with gut, skin, and excretory system bacteria, as well as environmental contaminants. Wet markets also contribute to the problem by frequently having lax sanitary conditions, with animals being stored in cramped spaces with poor ventilation and inconsistent hygiene practices [14].

**Figure 3:**
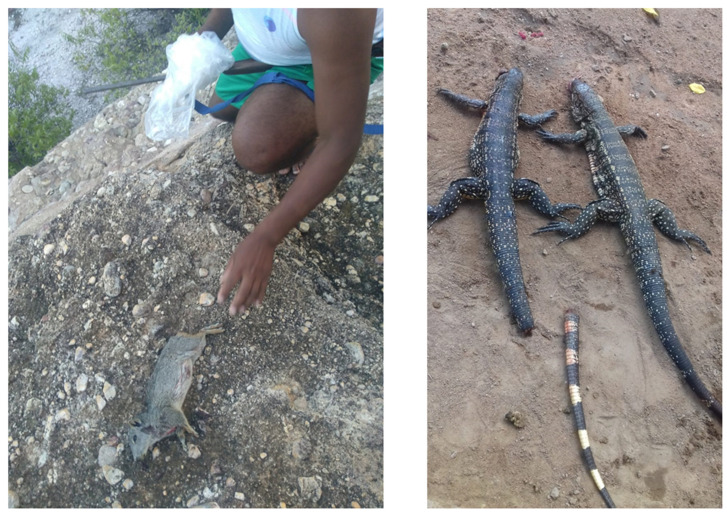
Mammals and reptiles are the most hunted species for consumption. (**Left**) Specimen of *Kerodon rupestris*, a rodent found in the Caatinga biome, and (**Right**) lizard of the genus *Tupinambis* sp. hunted and sold at an open market in a Brazilian city. Photo: Ianei Carneiro (**Left**) and Poliana Mascarenhas (**Right**).

Animal trafficking is also a strong potential source of mammal-to-mammal zoonotic infection [55]. Commonly consumed species, in particular mammals, are known for carrying zoonotic pathogens (Figure 3) [10]. Bird-to-human contamination in pet bird commercialization has also been detected recently and makes rearing and captivity breeding of wild birds a potential source of risk [56]. Mammals are a group of particular concern, as phylogenetic proximity is a contributor to pathogen spillover, likely due to similarities in physiology and immunity [57]. Contact between pets, livestock, and wildlife—in particular, if pets are involved in activities that enhance contact with either of the groups, such as herding or hunting, and are present during processing activities—is commonly one of the stages of zoonotic spillover, with pathogens brought closer to human populations after their establishment in livestock [14]. Hunting and transportation itself is a potential amplifier of zoonotic risk, as the stress experienced by the animal during hunting and transport (if alive) can be linked to immunosuppression and changes to pathogen shedding [58].

## 5. Current Evidence for Brazil

In spite of its potential importance to public health and the ubiquity of the practice of bushmeat consumption, studies investigating the link between the practice and zoonotic transmission in Brazil are still scarce. A recent review identified 173 parasite species interacting with 63 species of mammals targeted by hunting activities [59]. There is evidence of game meat being linked to 32 confirmed cases of infection by *Trypanosoma cruzi* [60]. Armadillos (genera *Dasypus* and *Euphractus*) are recognized as reservoirs for *Mycobaterium leprae*, *Trypanosoma* sp., *Paracoccidioides brasiliensis*, and *Leishmania* sp. [61,62], with the consumption of armadillos being associated with higher titers of anti-PGL-I (the *M. leprae*-specific antibody) in Amazonian populations [63]. However, a causal nexus between the consumption of armadillos and the incidence of leprosy in humans is yet to be firmly established [64]. Feral pigs/boars (*Sus scrofa*) are reservoirs of the tuberculosis bacterium, and there is evidence of an association between feral boar hunting practices and the acquisition of *Toxoplasma* infection [65,66]. Consuming the liver of wild ungulates (*Pecari* or *Tayassu* sp.) was detected as a risk factor for parasitism by the helminth *Calodium hepaticum* [67].

## 6. Final Considerations

Current trends in the sociopolitical and ecoepidemiological dynamics indicate that the risk of the emergence of pathogens with pandemic potential is high [68]. Thirteen and a half million Brazilians, or 6.4% of the national population, live in conditions of extreme poverty according to data from 2019 [69], with the majority of this contingent concentrated in the northern and northeastern regions—the regions most affected by the COVID-19 pandemic [70]. In the same regions, ethnobiological studies highlight the close bond and reliance of these populations on ethnobiological and ethnomedical applications of the fauna, suggesting that the most socioeconomically marginalized portion of the population is also highly vulnerable and more heavily taxed by zoonotic disease [71]. The impacts of disease are exacerbated by extreme economic and environmental poverty, ecosystem erosion, and human-accelerated climate change. This changing scenario is highly concerning given the importance of game species for the survival and subsistence of populations that are particularly sensitive to these changes which, in turn, could further increase their risk of exposure to zoonotic sources [72]. Forest suppression increases local temperatures and facilitates the spread of zoonotic and vector-borne diseases [73,74]. The current scenario indicates the high risk of the emergence of novel public health threats and potential Public Health Emergencies of International Concern (PHEIC) in the context of wildlife consumption in Brazil, highlighting the necessity of the One Health approach that allows for the survival and subsistence of such populations, enable the protection of ecosystems and biodiversity services, and mitigate the risks of establishing novel and known wildlife-borne pathogen transmission.

## Supplementary Materials

The following supporting information can be downloaded at: https://www.mdpi.com/article/10.3390/pathogens13030222/s1, Table S1: List of animal species consumed in Brazil, classified by major taxonomic groups, as observed in literature. Species names are as reported in the references. Species in bold lettering are domestic/introduced species. Species without an assigned usage in the table either have no clear usage expressed in their publication of origin, or are reported to be bred/kept in captivity; Table S2: Sources of ethnobiological data explored in the manuscript. References [75,76,77,78,79,80,81,82] are cited in Table S1.
