# Supplementary material for: Wildlife as Food and Medicine in Brazil: A Neglected Zoonotic Risk?
Source: Pathogens. 2024 Mar 2;13(3):222. doi: 10.3390/pathogens13030222 (PMC10975579; doi:10.3390/pathogens13030222)
Supplement: Supplementary file 1 [file pathogens-13-00222-s001.zip › Table S1.pdf]

Table S1. List of animal species consumed in Brazil, classified by major taxonomic groups, as observed in literature. Species names are as reported in the references. Species in bold lettering are domestic/introduced species. Species without an assigned usage in the table either have no clear usage expressed in their publication of origin, or are reported to be bred/kept in captivity.

Clade	Order	Species	Medicine	Food	Type of product	References
**Aves**	Accipitriformes	*Buteo brachyurus*		X		[12]
		Buteo nitidus		X		[12]
		Buteogallus urubitinga	X		Bones	[38]
		Geranoaetus melanoleucus		X		[12]
		Heterospizias meridionalis		X		[12]
		Rupornis magnirostris		X		[12,22]
	Anseriformes	***Anas platyrhynchos***	X		whole animal, eggs	[38,75,29,36]
		Anhima cornuta	X			[21]
		Anser anser	X	X		[21,10]
		Dendrocygna viduata		X		[12]
		Netta erythrophthalma	X		Eggs	[38]
		Sarkidiornis sylvicola	X	X	Caruncle	[12,13]
	Apodiformes	"Hummingbird"	X		Whole animal, nest	[75]
		Amazilia fimbriata		X		[22]
		Anopetia gounellei		X		[22]
		Chlorostilbon lucidus	X		Nest	[13]
		Eupetomena macroura	X	X		[22,21]
		Phaethornis pretrei		X		[22]
		Thalurania watertonii		X		[22]
	Caprimulgiformes	*Chordeiles acutipennis*		X		[12]
		Hydropsalis albicollis		X		[22]
		Nyctidromus albicollis	X			[21]
	Nyctiibiformes	*Nyctibius griseus*	X			[12,22]
	Cariamiformes	*Cariama cristata*	X	X	Feathers, fat	[12,22,76,24]
	Cathartiformes	*Cathartes aura*				[12]
		Coragyps atratus	X		Liver, feathers, beak	[12,38,75,29,21,76,24,49,18,40,41, 31]
	Charadriiformes	*Jacana jacana*		X		[12]
		Vanellus chilensis	X	X		[12,21]

Ciconiiformes	*Ciconia maguari*	X	X		[21,10]
Columbiformes	*Claravis pretiosa*		X		[12,22]
	Columba livia	X	X	Meat	[38,21,10]
	Columbina minuta	X	X	Feet, feces, meat	[12,22,13,76]
	Columbina picazuro	X		Whole animal	[38]
	Columbina picui	X	X	Feet, feces, meat	[12,22,13,76]
	Columbina squammata	X	X	Feet, feces, meat	[12,22,13]
	Columbina talpacoti	X	X	Feet, feces, meat	[12,22,13,76]
	Leptotila rufaxilla	X	X	Meat, gizzard, membrane, whole animal	[38,21,10,13]
	Leptotila verreauxi	X	X	Meat	[12,22,76]
	Patagioenas picaruzo		X		[12]
	Zenaida auriculata		X		[12]
Coraciiformes	*Chloroceryle amazona*				[12]
Cuculiformes	*Coccyzus melacoryphus*				[12]
	Crotophaga ani	X	X	Feathers, whole animal, meat, flesh	[12,22,21,13,24,18,31]
	Guira guira	X	X		[12,22,21]
	Piaya cayana		X		[22]
	Tapera naevia		X		[22]
Falconiformes	*Caracara plancus*	X	X		[12]
	Falco rufigularis	X			[21]
	Falco femoralis				[22]
	Herpetotheres cachinnans	X		Whole animal	[12,38,21]
Galbuliformes	*Galbula ruficauda*		X		[22]
	Nystalus maculatus		X		[12]
Galliformes	***Coturnix coturnix***	X		Eggs	[38]
	Gallus gallus	X		Fat, eggs, gizzard, esophagus, meat, feathers	[38,75,29,36,21,24,49,18,40,41,31,77,30,26,37]
	Meleagris gallopavo	X	X	Feathers, fat	[75,29,21,10,24,49,77]
	Numida meleagris	X		Fat, blood, meat	[38,75,29,36,40]
	Pauxi tuberosa	X		Beak, gizzard	[26]
	Pavo cristatus	X		Feathers	[38,75,29,21,40,41,31]

		Penelope jacucaca	X	X	Feathers	[21,10,13]
		Penelope superciliaris		X		[22]
	Gruiformes	*Aramides cajaneus*	X	X		[12,21]
		Aramus guarauna		X		[12]
		Gallinula galeata		X		[12]
		Psophia viridis	X		Leg	[26]
	Nyctiibiformes	*Nyctibius griseus*	X			[12,22]
	Passeriformes	*Agelaioides fringillarius*		X		[12]
		Ammodramus humeralis				[12,36]
		Chrysomus ruficapillus				[39]
		Cnemotriccus fuscatus		X		[22]
		Coereba flaveola	X	X		[22,21,39]
		Coryphospingus pileatus				[12,39]
		Cyanocorax cyanopogon	X	X	Feathers, whole animal, gizzard	[12,38,22,75,36,21,13,76,24,40,39]
		Cyanoloxia brissonii		X		[12,22,39]
		Dacnis cayana				[22]
		Dysithamnus mentalis		X		[22]
		Elaenia chiriquensis		X		[22]
		Elaenia cristata		X		[22]
		Elaenia flavogaster		X		[22]
		Elaenia mesoleuca		X		[22]
		Elaenia parvirostris		X		[22]
		Empidonomus varius		X		[22]
		Estrilda astrild				[39]
		Euphonia chlorotica		X		[12,22,39]
		Euscarthmus meloryphus		X		[22]
		Fluvicola nengeta	X	X	Whole animal	[12,38,21]
		Formicivora grisea		X		[22]
		Formicivora melanogaster		X		[22]
		Furnarius rufus	X			[21]
		Furnarius leucopus				[12]
		Gnorimopsar chopi		X		[12,39]

	Hemitriccus margarita-ceiventer		X		[22]
	Herpsilochmus atricapillus		X		[12,22]
	Herpsilochmus longirostris		X		[22]
	Icterus cayanensis				[12]
	Icterus jamacaii		X		[12,22,39]
	Icterus pyrrhopterus				[12,22,39]
	Lanio cucculatus				[22]
	Lanio pileatus				[22]
	Lathrotriccus euleri		X		[22]
	Legatus leucophaius		X		[22]
	Lepidocolaptes angustirostris		X		[22]
	Leptopogon amaurocephalus		X		[22]
	Megarynchus pitangua		X		[22]
	Mimus saturninus	X	X	Meat	[12,22,76,39]
	Molothrus bonariensis				[12]
	Myiarchus ferox		X		[22]
	Myiarchus swainsoni		X		[22]
	Myiarchus tyrannulus		X		[22]
	Myiobius atricaudus		X		[22]
	Myiodynastes maculatus		X		[22]
	Myiopagis caniceps		X		[22]
	Myiopagis viridicata		X		[22]
	Myiophobus fasciatus		X		[22]
	Myrmorchilus strigilatus		X		[22]
	Paroaria dominicana	X	X		[12,22,39]
	Passer domesticus				[12]
	Phacellodomus rufifrons		X		[22]
	Pitangus sulphuratus		X		[12,22,39]
	Polioptila plumbea		X		[12,22]
	Procacicus solitarius				[12]
	Progne chalybea	X		Whole animal	[12]
	Pseudoseisura cristata				[12]
	Sakesphorus cristatus		X		[22]

		Saltator similis			[39]
		Schistochlamys ruficapillus			[22]
		Sclerurus scansor		X	[22]
		Sicalis flaveola		X	[12,22,39]
		Sicalis luteola			[39]
		Spinus yarrellii			[12,39]
		Sporophila albogularis		X	[12,22]
		Sporophila angolensis			[12,39]
		Sporophila ardesiaca			[12,39]
		Sporophila bouvreuil			[39]
		Sporophila leucoptera			[12]
		Sporophila lineola			[12,22]
		Sporophila nigricollis			[12,22]
		Sturnella superciliaris			[12]
		Tachyphonus rufus			[12]
		Tangara cayana		X	[22]
		Tangara palmarum		X	[22]
		Tangara sayaca		X	[22]
		Taraba major		X	[22]
		Thamnophilus capistratus		X	[12,22]
		Thamnophilus punctatus		X	[22]
		Thamnophilus torquatus		X	[22]
		Troglodytes musculus		X	[12]
		Turdus albicollis			[22]
		Turdus amaurochalinus			[12,22]
		Turdus leucomelas		X	[12,22]
		Turdus rufiventris		X	[12,22]
		Tyrannus melancholicus		X	[22]
		Volatinia jacarina		X	[12]
		Xolmis dominicanus			[12]
		Zonotrichia capensis		X	[12,22]
	Pelecaniformes	*Ardea alba*		X	[12]
		Ardea cocoi	X	X	[21,10]

		Egretta thula		X		[12]
		Tigrisoma lineatum		X		[12]
	Piciformes	"acari"	X		Skin	[18]
		Celeus flavescens		X		[22]
		Dryocopus lineatus	X			[21]
		Piculus chrysochloros		X		[22]
		Pteroglossus aracari	X			[21]
		Pteroglossus inscriptus	X			[21]
		Ramphastos tucanus	X		Beak	[21,18]
		Ramphastos vitellinus	X			[21]
		Veniliornis passerinus		X		[12,22]
	Podicipediformes	*Tachybaptus dominicus*	X		Gizzard, membrane	[13]
	Psittaciformes	*Amazona aestiva*	X	X	Feces	[12,38,22]
		Amazona sp.	X		Feces	[49]
		Anodorhynchus hyacinthinus	X		Beak	[26]
		Ara araruna	X		Beak	[26]
		Ara chloropterus	X		Beak	[26]
		Ara macao	X		Beak	[26]
		Eupsittula cactorum	X		Meat	[76]
		Eupsittula cactorum		X		[12,22]
		Forpus xanthopterygius		X		[12,22]
	Rheiformes	***Rhea americana***	X	X	Fat, eggs	[38,29,21,10,24,18,40]
	Strigiformes	*Glaucidium brasilianum*				[12,22]
		Megascops choliba				[12]
		Rhynchotus rufescens	X	X		[12,21,10]
	Struthioniformes	*Struthio camelus*	X		Toasted egg shells	[75,78]
	Tinamiformes	*Crypturellus noctivagus*	X	X		[22,21,10]
		Crypturellus parvirostris		X		[12,22]
		Crypturellus tataupa	X	X		[12,22]
		Nothura boraquira	X	X	Feathers	[12,21,10,13,76]
		Nothura maculosa cearensis	X	X	Feathers	[22,75,29,13,24,77,30,37]
		Nothura spp.		X		[13]
		Tinamus tao	X		Feet scales, feathers, head	[26]

	Trogoniformes	*Trogon curucui*		X		[22]
**Non-avian Dyapsida**	Crocodyla	*Crocodylus sp.*	X		Skin, bone	[32]
		Caiman crocodilus	X	X	Skin, teeth, fat	[29,21,10,18,40,31,26,25,33]
		Caiman latirostris	X	X	Leather, fat, skin	[75,21,10,24,49,18,40,25,33]
		Caiman sp.	X		Skin, bone	[32]
		Caiman yacare	X	X		[25]
		Melanosuchus niger	X	X	Fat, skin, penis	[21,10,18,33]
		Paleosuchus palpebrosus	X	X	Leather, fat, penis, tooth	[21,10,49,18,40,25,33]
		Paleosuchus sp.	X		Skin, bone	[32]
		Paleosuchus trigonatus	X	X		[21,10,25]
	Squamata	*Ameiva ameiva*	X	X		[21,25,33]
		Anolis fuscoauratus	X			[33]
		Boa constrictor	X	X	Fat	[26,21,10,13,49,18,40,25,33]
		Bothrops erythromelas	X		Whole animal	[13]
		Bothrops leucurus	X			[21,33]
		Bothrops sp.	X		Fat, whole animal, powdered product	[49,18,78,32]
		Caudisona durissa	X	X	Fat	[24,25]
		Chironius sp.	X		Powdered product	[32]
		Cnemidophorus ocellifer	X	X		[36,21,25]
		Corallus caninus	X		Whole animal	[36,21,25]
		Corallus hortulanus	X			[36,21,25]
		Crotalus durissus	X	X	Rattle, fat, tooth, skin	[38,75,29,36,21,13,40,41,31,77,30, 32,25]
		Dracaena guianensis		X		[25]
		Epicrates assisi	X			[36,25]
		Epicrates cenchria	X		Fat	[29,21,49,18,25]
		Eunectes murinus	X	X	Fat	[29,21,10,49,18,40,26,25]
		Eunectes notaeus	X	X		[25]
		Hemidactylus mabouia	X		Whole animal	[29,36,21,49,18,25]
		Iguana iguana	X	X	Bone, fat, lung, leather, meat, whole animal, tail	[38,75,36,21,10,13,24,40,41,77,30, 37,32,25]

		Lachesis muta	X	X	Fat	[21,49,18,25]
		Leptophis ahaetulla	X		Whole animal	[21]
		Liophis sp.	X		Powdered product	[32]
		Mastigodryas bifossatus	X			[21,25]
		Micrurus ibiboboca	X			[36,21,25]
		Micrurus sp.	X		Fat, whole animal	[18]
		Oxyrhopus guibei	X			[25]
		Oxyrhopus petola	X			[25]
		Oxyrhopus trigeminus	X		Fat, whole animal	[36,21,30,40,25]
		Philodryas nattereri				[25]
		Phyllopezus pollicaris	X			[36]
		Placosoma sp.	X		Skin	[32]
		Polychrus acutirostris	X			[36,25]
		Polychrus marmoratus	X			[21,25]
		Salvator merianae	X		Leather, liver, fat	[13]
		Spilotes pullatus	X		Whole animal	[36,21,18,25]
		Tropidurus hispidus	X		Whole animal, bone, leather, liver, viscera	[38,36,21,13,49,18,31,78,25,33]
		Tropidurus semitaeniatus	X			[21,25]
		Tropidurus torquatus	X			[21,25]
		Tupinambis merianae	X	X	Fat, meat	[38,29,36,21,10,24,18,41,31,77,30,78,25,33]
		Tupinambis rufescens	X	X		[25]
		Tupinambis sp.	X		Fat, tongue, skin	[49]
		Tupinambis teguixin	X	X	Fat, tail	[21,10,18,26,25]
		Uranoscodon superciliosus	X			[21,25]
		Xenodon merremii				[25]
	Testudines	*Caretta caretta*	X	X	Fat	[21,10,49,25]
		Chelonia mydas	X	X	Fat	[21,10,49,25]
		Chelonoidis carbonarius	X	X	Fat, whole animal	[38,36,24,26,25]
		Chelonoidis denticulata	X	X	Carapace, whole animal, fat, liver, urine	[75,29,40,26,25]
		Chelus fimbriatus	X	X		[25]
		Dermochelys coriacea	X	X	Fat, oil from skin	[21,10,49,25]

		Eretmochelys imbricata	X	X	Fat	[21,10,49]
		Geochelone carbonaria	X		Carapace, fat, liver, blood	[21,49,79]
		Geochelone denticulata	X		Carapace, fat, liver	[21,79]
		Kinosternon scorpioides	X	X		[36,25]
		Lepidochelys olivacea	X	X		[21,10,49,25]
		Mesoclemmys nasuta		X		[25]
		Mesoclemmys tuberculata	X	X	Fat, meat, eggs	[38,36,21,13,25]
		Peltocephalus dumerilianus	X	X		[21,10,25,43]
		Phrynops geoffroanus	X	X	Carapace, fat, hoof	[75,59,36,21,24,49,18,40,37,25]
		Phrynops spp.	X		Fat	[77]
		Phrynops tuberosus	X	X	Fat, shell, blood, eggs	[36,13,49,48,41,31,25]
		Platemys platycephala		X		[25]
		Podocnemis erythrocephala	X	X		[25]
		Podocnemis expansa	X	X	Fat	[21,10,25,79,43]
		Podocnemis sextuberculata	X	X		[21,10,25,43]
		Podocnemis unifilis	X	X		[21,10,25]
		Rhinoclemmys punctularia	X	X		[21,10,25]
**Mammalia**	Carnivora	***Canis lupus familiaris***	X		Feces, head	[38,36,21,49]
		Cerdocyon thous	X		Fat, bone, liver, meat, tail, leather, suet	[38,75,21,13,24,49,18,40,41,31,77,30,37,11]
		Chrysocyon brachyurus	X			[21]
		Conepatus amazonicus	X	X		[11]
		Conepatus semistriatus	X	X	Bones, fat, meat, tail, scent glands	[38,75,29,36,21,10,13,24,40]
		Conepatus sp.	X		Bone	[49]
		Dusicyon thous	X			[21]
		Eira barbara	X			[11]
		Felis catus	X		Whole animal	[38]
		Felis silvestris	X		Fur	[31]
		Galictis cuja		X		[11]
		Leopardus pardalis	X		Tail, fat	[13,11]
		Leopardus tigrinus	X		Meat, tail, fat, leather	[13,24,11]

	Leopardus wiedii	X			[11]
	Lontra longicaudis	X			[21,10]
	Nasua nasua	X	X	Penis, fat, bone	[21,49,18,11]
	Panthera onca	X			[21,26,11]
	Procyon cancrivorus	X		Fat, skin,tail, leather	[21,13,24,49,18,40,31,11]
	Puma concolor	X		Fat	[21,13,11]
	Puma yagouaroundi	X		Fat, tail, leather	[13,24,11]
	Speothos venaticus	X			[21]
	"whale"	X		Fat	[24]
	Balaenoptera acutorostrata	X		Fat, oil	[24,49,18]
Cetartiodactyla	*Blastocerus dichotomus*	X			[21,10]
	Bos taurus	X		Bezoar, milk, horn, butter, liver, skull marrow, urine, penis, hoof	[38,75,29,36,21,24,18,40,41,31,77,30,37]
	Bubalus bubalis	X	X	Horn, fat	[21,10,18]
	Capra hircus	X	X	Milk, horn, hair	[38,21,10,24,18,41,77]
	Inia geoffrensis	X	X	Fat, oil from skin	[21,10,49,18]
	Mazama americana	X	X	Marrow, fat, bowel fat	[21,10,26,11]
	Mazama gouazoubira	X	X	Horn, fat	[21,10,24,11]
	Mazama simplicicornis	X	X		[21,10]
	Mazama sp.	X		Horn, hoof, tail, tibia	[18,41]
	Ovis aries	X		Fat, castrated ram suet, horn, skull, leather	[2,75,29,36,21,24,49,18,40,41,31,77,37]
	Ozotoceros bezoarticus	X	X		[21,10]
	Pecari tajacu	X	X	Bowels, testicles, fat	[21,10,26,11]
	Physeter catodon	X		Fat	[21,18]
	Physeter macrocephalus	X		Fat, oil	[49]
	Sotalia fluviatilis	X	X	Fat, oil from skin	[21,10,49,18]
	Sotalia guianensis	X	X	Fat, oil from skin	[21,10,49,18]
	Sus scrofa domesticus	X		Fat, navel, fat from scrotum, testicle	[38,21,24,49,18,41,31,77,30,37]
	Tayassu pecari	X	X	Teeth, testicles	[21,10,26,11]

	Tayassu tajacu	X		Fat	[18]
Chiroptera	*Molossus molossus*	X		Whole animal	[38]
Cingulata	*Cabassous tatouay*	X	X		[11]
	Dasypus novemcinctus	X	X	Fat, skin, tail, paws, meat, liver	[38,29,36,21,10,13,24,49,18,41,31,11]
	Dasypus septemcinctus	X	X		[11]
	Dasypus sp.	X		Fat, urine, tail, nails	[26]
	Euphractus sexcinctus	X	X	Fat, skin, tail, paws, meat, urine	[75,29,21,10,13,24,49,18,40,51,31,77,30,11]
	Priodontes maximus	X	X	Nails, fat, tail	[26,11]
	Tolypeutes sp.	X		Skin, fat, tail	[18,32]
	Tolypeutes tricinctus	X	X		[21,10,11]
Didelphimorphia	*Didelphis marsupialis*	X	X	Fat	[21,49,18,11,80]
	Didelphis karkinophaga	X	X		[11]
	Didelplhis albiventris	X	X	Bones, meat, fat	[38,29,21,13,49,80]
Lagomorpha	*Sylvilagus brasiliensis*	X	X	Fur, feces	[21,10,49,26,11]
Perissodactyla	***Equus asinus***	X		Milk, hoof, foot-prints	[38,75,29,21,18,40,41]
	Equus caballus	X		Hair	[38,21]
	Tapirus terrestris	X	X	Fat, penis, nails	[21,10,18,26,11]
Pilosa	*Bradypus sp.*	X		Claw, skin	[49]
	Bradypus tridactylus	X	X		[10,18]
	Bradypus variegatus	X	X	Claw, skin, bone, fat	[29,21,10,18,40,11]
	Myrmecophaga tetradactyla	X	X		[21,10]
	Myrmecophaga tridactyla	X	X	Tail hair	[21,10,26,11]
	Tamandua tetradactyla	X	X	Meat, leather, fat, bones, claws	[13,24,49,11]
Primate	*Callicebus barbarabrownae*				[11]
	Alouatta belzebul	X	X		[22,21,10,11]
	Alouatta nigerrima	X	X		[22,21,10]
	Alouatta seniculus	X	X		[22,21,10]
	Callithrix jacchus	X	X		[11]
	Sapajus apella	X	X	Penis, bone, fat	[21,10,18]
	Sapajus libidinosus	X	X		[11]

		Sapajus libidinosus	X		Meat	[13]
	Rodentia	*Agouti paca*	X	X	Fat, bile	[21,10,24,18]
		Cavia aperea	X	X	Fat, teeth, head, whole animal	[38,21,10,24]
		Coendou baturitensis	X	X		[11]
		Coendou prehensilis	X	X	Spine	[75,29,21,10,49,18,40,11]
		Cuniculus paca	X	X	Gallbladder, bile	[13,26,11]
		Dasyprocta prymnolopha	X	X		[21,10,11]
		Galea spixii	X	X	Meat, teeth, bones	[13,11]
		Hydrochoerus hydrochaeris	X	X	Bone, fat, skin, flesh	[29,36,21,10,18,26,32,11]
		Kerodon rupestris	X	X	Fat, manure, coalho (part of stomach), meat, feces, fel, gallbladder, bones	[38,75,36,21,10,13,24,11]
		Trichomys laurentius	X		Feces	[13,11]
		Trichechus inunguis	X	X	Fat, skin	[38,29,21,10,49,18]
	Sirenia	*Trichechus manatus*	X	X	Fat, skin, milk	[36,21,10,49,18,40]
**Amphibia**	Anura	"frogs"	X		Fat	[29,18]
		Bufo sp.	X		Whole animal	[49,18]
		Chaunus marinus	X			[21]
		Chaunus schneideri	X			[21]
		Leptodactylus labyrinthicus	X		Fat	[21,33]
		Leptodactylus sp.	X		Fat	[18]
		Leptodactylus vastus	X		Meat, fat	[38,13]
		Rhinella jimi	X		Fat, leather, guts, secretions, abdomen	[75,29,36,13,33]
		Rhinella marina	X		whole animal (in powder), Abdomen (live animal)	[26]
		Rhinella schneideri	X		Viscera, fat	[24]
		Rhinella sp.	X		Whole animal	[32]
		Trachycephalus resinifictrix	X		Pitch	[26]
**Sharks and Rays**	Carchahiniformes	*Carcharhinus leucas*	X			[27]
		Carcharhinus limbatus	X		Cartilage, fat	[21]
		Carcharhinus porosus	X	X	Cartilage, fat	[21,10,49,27]

		Galeocerdo cuvier	X	X	Cartilage, fat	[21,10,49,27]
		Rhizoprionodon lalandii	X	X	Cartilage, oil, fat	[21,10,49,27]
		Rhizoprionodon porosus	X	X	Cartilage, oil, fat	[21,10,49,27]
		Sphyrna lewini	X	X	Liver oil, bile	[21,10,27]
		Sphyrna sp.	X		Cartilage	[49,18]
	Myliobatiformes	"ray fish"	X		Flesh, tail, fat	[29,79]
		Aetobatus narinari	X	X	Tooth, liver oil, tail, mucous from ventral region, liver	[21,10,49,27]
		Dasyatis guttata	X	X	Tooth, liver oil, tail, mucous from ventral region, liver	[21,10,27]
		Dasyatis marianae	X	X	Tooth, liver oil, tail, mucous from ventral region, liver	[21,10,49,27]
		Dasyatis sp.	X		Tail, fat	[79]
		Paratrygon aiereba	X	X		[21,10]
		Plesiotrygon iwamae	X	X	Fat	[21,10,27]
		Potamotrygon histrix	X	X	Spur, fat	[21,10,27]
		Potamotrygon motoro	X	X	Spur, fat	[21,10,27]
		Potamotrygon orbignyi	X	X	Fat	[21,10,27]
		Potamotrygon sp.	X		Spur	[26]
		Urotrygon microphthalmum	X	X	Tooth, liver oil, tail, mucous from ventral region, liver	[21,10,49,27]
**Bony fish**	Orectolobiformes	*Ginglymostoma cirratum*	X	X	Cartilage, oil	[21,10,49,27]
	Rajiformes	*Atlantoraja cyclophora*	X	X	Eggs	[21,10,27]
	Rhinopristiformes	*Pristis pectinata*	X	X	Rostrum	[21,10,18,27]
		Pristis perotteti	X	X	Rostrum ("sword")	[21,10,18,27]
	Torpediniformes	*Narcine brasiliensis*	X	X	Fat	[21,10,27]
	Anguiliformes	*Gymnothorax funebris*	X	X	Flesh	[21,10,49,27]
		Gymnothorax moringa	X	X	Flesh	[21,10,49,27]
		Gymnothorax vicinus	X	X	Flesh	[21,10,49,27]
	Batrachoidiformes	*Thalassophryne nattereri*	X	X	Flesh, eye, brain	[21,10,49,27]
	Beloniformes	*Exocoetus sp.*	X		Fat	[49]

	Beryciformes	*Holocentrus adscensionis*	X	X	Sting	[21,10,49,79,27]
	Carangiformes	*Echeneis naucrates*	X	X		[21,10]
	Characiformes	*Astyanax bimaculatus*	X	X	Whole animal	[21,10,27]
		Brycon nattereri	X	X	Meat	[21,10,27]
		Chalceus macrolepidotus	X		Whole animal, eye	[27]
		Colossoma macropomum	X	X		[21,10]
		Erythrinus erythrinus	X	X	Whole animal	[21,10,27]
		Hoplias aimara	X		Fat	[26]
		Hoplias lacerdae	X		Fat	[27]
		Hoplias malabaricus	X	X	Dermal secretion, fat, head, meat, guts, scale, whole animal	[75,29,21,10,24,49,18,41,31,27]
		Hydrolycus scomberoides	X	X	Fat	[21,10,27]
		Leporinus friderici	X	X		[21,10]
		Leporinus piau	X		Fat	[27]
		Leporinus steindachneri	X		Fat	[31,27]
		Mylossoma duriventre	X	X	Fat	[21,10,27]
		Paracheirodon axelrodi	X		Whole animal	[27]
		Piaractus brachypomus	X		Fat	[27]
		Prochilodus argenteus	X	X		[21,10]
		Prochilodus nigricans	X	X	Fat, gall, meat	[21,10,41,31,27]
		Prochilodus spp.	X		Fat	[24]
		Salminus hilarii	X		Head	[27]
		Schizodon knerii	X	X	Fat	[21,10,27]
		Serrasalmus brandtii	X	X	Tail, gall, fat	[21,10,27]
	Cichliformes	*Cichla melaniae*	X		Caudal fin	[26]
	Clupeiformes	*Opisthonema oglinum*	X	X	Whole animal	[21,10,27]
	Elopiformes	*Megalops atlanticus*	X	X	Scale	[21,10,49,79,27]
	Gadiformes	*Gadus morhua*	X	X	Fat, skin	[38,21,10,27]
	Gymnotiformes	*Electrophorus electricus*	X		Fat, spine, whole animal, bone	[29,21,24,49,40,31,79,27]
	Lophiiformes	*Ogcocephalus vespertilio*	X		Whole animal	[21,49,27]
	Osteoglossiformes	*Arapaima gigas*	X	X	Scale	[21,10,27]
		Osteoglossum ferreirai	X		Scale	[27]

	Perciformes	*Calamus penna*	X	X	Fin	[21,10,27]
		Centropomus parallelus	X	X		[21,10]
		Centropomus undecimalis	X	X	Fat	[21,10,27]
		Cynoscion acoupa	X	X	Otolith	[21,10,27]
		Cynoscion leiarchus	X	X	Otolith	[21,10,27]
		Micropogonias furnieri	X	X	Otolith	[21,10,27]
		Pachyurus francisci	X	X	Otolith	[21,10,27]
		Plagioscion squamosissimus	X	X	Otolith	[21,10,27]
		Plagioscion surinamensis	X	X	Otolith	[21,10,27]
		Trichiurus lepturus	X	X	Tail	[21,10,27]
	Scorpaeniformes	*Scorpaena sp.*	X		Flesh	[49]
	Siluriformes	*Aspistor luniscutis*	X		Whole animal	[27]
		Aspredinichthys tibicen	X	X	Barbels	[21,10,49,27]
		Aspredo aspredo	X	X	Barbels	[21,10,49,27]
		Bagre bagre	X	X	Whole animal	[21,10,27]
		Brachyplatystoma filamento-sum	X		Fin	[27]
		Callichthys callichthys	X	X	Whole animal	[21,10,27]
		Cetopsis candiru	X		Meat	[27]
		Franciscodoras marmoratus	X	X		[21,10]
		Genidens barbus	X	X	Whole animal	[21,10,27]
		Genidens genidens	X	X	Whole animal	[21,10,27]
		Lithodoras dorsalis	X	X	Fat	[21,10,27]
		Megalodoras uranoscopus	X	X	Fat	[21,10,27]
		Oxydoras niger	X	X	Fat	[21,10,27]
		Phractocephalus hemioliop-terus	X	X	Fat	[21,10,27]
		Pimelodella brasiliensis	X	X		[21,10]
		Platydoras costatus	X	X		[21,10]
		Pseudoplatystoma corruscans	X	X	Fat	[21,10,27]
		Pseudoplatystoma fasciatum	X	X	Fat, gall	[21,10,27]
		Pterodoras granulosus	X	X	Fat	[21,10,27]
		Sciadeichthys luniscutis	X	X		[21,10]
		Sorubimichthys planiceps	X	X	Meat	[21,10,27]

		Trachelyopterus galeatus	X	X	Whole animal, spur	[21,10,27]
		Zungaro zungaro	X		Fat, skin, meat	[21,27]
	Synbranchiformes	*Synbranchus marmoratus*	X	X	Whole animal	[21,10,27]
	Syngnathiformes	*Hippocampus erectus*	X		Whole animal	[21,27]
		Hippocampus reidi	X		Whole animal	[75,29,21,49,31,79,27]
	Tetraodontiformes	*Balistes capriscus*	X	X	Skin	[21,10,27]
		Balistes vetula	X	X	Skin, tooth	[21,10,49,18,27]
		Colomesus psittacus	X	X	Liver oil, bile	[21,10,49,27]
		Sphoeroides testudineus	X	X	Fat	[21,10,27]
**Hexapoda**	Blattodea	"termite"	x		Whole animal	[41]
		Eurycotis manni	X			[21]
		Microcerotermes exiguus	X			[21]
		Nasutitermes corniger	X		Whole animal	[24,81]
		Nasutitermes macrocephalus	X		Whole animal	[38,75,29,36,40]
		Periplaneta americana	X		Whole animal	[38,75,29,21,49,40,31,32]
	Coleoptera	"beetle"	X		Nest	[36]
		Coraliomela brunnea	X		Whole animal, meat	[29,21,18]
		Curculionidae	X		Larvae	[31]
		Pachymerus nucleorum	X			[21]
		Palembus dermestoides	X			[21]
		Rhinostomus barbirostris	X			[21]
		Rhynchophorus palmarum	X			[21]
	Diptera	*Musca domestica*	X		Whole animal	[75,29,21]
	Hemiptera	*Abedus sp.*	X		Whole animal	[32]
		Belostoma sp.	X		Whole animal	[32]
		Diplonychus sp.	X		Whole animal	[32]
	Hymenoptera	"wasp"	X		Whole animal	[18]
		Acromyrmex landolti	X		Whole animal	[38]
		Apis mellifera	X		Honey	[38,75,29,36,21,24,49,18,40,26]
		Apoica pallens	X			[21]
		Atta cephalotes	X		Abdomen	[38,21,49]
		Atta sexdens	X		Anthill	[32]
		Brachygastra lecheguana	X			[21]

		Cephalotrigona capitata	X		Honey	[36,21]
		Frieseomelitta silvestrii	X			[21]
		Frieseomelitta varia	X		Honey	[75,29,79]
		Melipona compressipes	X		Honey	[29,21,49,18]
		Melipona mandacaia	X			[21]
		Melipona scutellaris	X		Honey	[75,29,21,49,18,40,41,31]
		Melipona sp.	X		Honey	[29,18]
		Melipona quadrifasciata	X			[21]
		Melipona subnitida	X		Honey	[75,29,36,21,24,18,41,31]
		Partamona cupira	X		Honey, bee wax	[75,29,36,21,41,31]
		Partamona seridoensis	X		Honey, "saburá"	[24]
		Plebeia emerina	X			[21]
		Polistes canadensis	X		Sting	[21,77]
		Polybia sericea	X			[21]
		Pronectarina sylveirae	X		Nest	[38]
		Protopolybia exigua	X			[21]
		Scaptotrigona sp.	X		Honey	[75,29,24,18]
		Synoeca surinama	X			[21]
		Tetragonisca angustula	X		Honey	[75,29,36,21,18]
		Trigona mosquito	X			[21]
		Trigona spinipes	X		Honey	[75,29,21,41]
	Lepidoptera	*Oiketicus kirbyi*	X			[21]
	Orthoptera	"cricket"	X		Leg	[31]
		Acheta domesticus	X			[21]
		Gryllus assimilis	X		Leg	[41]
**Myriapoda**	Scolopendromorpha	*Scolopendra spp.*	X		Whole animal	[49,18]
**Chelicerata**	Araneae	*Tarantulas sp.*	X		Whole animal	[18]
	Scorpiones	*Bothriurus asper*	X		Sting	[24]
		Bothriurus sp.	X		Whole animal	[49]
		Rhopalurus rochai	X		Sting	[24]
**Crustacea**	Decapoda	*Aratus pisonii*	X	X	whole animal, flesh	[21,10,49]
		Calappa ocellata	X			[21]
		Cardisoma guanhumi	X	X		[21,10,82]

		Emerita portoricensis	X			[21]
		Goniopsis cruentata	X	X	Flesh, bile	[21,10,49]
		Litopenaeus schmitti	X		Fat	[49]
		Macrobrachium acanthurus	X	X	Flesh	[21,10,49]
		Macrobrachium borellii	X	X	Flesh	[21,10,49]
		Macrobrachium carcinus	X	X		[21,10]
		Macrobrachium sp.	X		Spines	[79]
		Ocypode quadrata	X		Carapace, bile	[21,49]
		Plagusia depressa	X			[21]
		Uca maracoani	X		Whole animal	[21,49]
		Ucides cordatus	X	X	Bile	[21,10,49,82]
		Xiphopenaeus kroyeri	X	X		[21,10,49]
		Xiphopenaeus schmitti	X	X		[10]
	Stomatopoda	*Cloridopsis dubia*	X			[21]
		Squilla sp.	X		Whole animal	[49]
**Echinodermata**	Clypeasteroida	*Encope sp.*	X		Whole animal	[49,79]
		Mellita quinquiesperforata	X		Whole animal	[29]
		Mellita sexiesperforata	X			[21]
	Paxillosida	*Astropecten sp.*	X		Whole animal	[29,49,79]
		Luidia senegalensis	X		Whole animal	[29,21,79]
	Spinulosida	*Echinaster brasiliensis*	X		Whole animal	[29,21]
		Echinaster echinophorus	X		Whole animal	[29,21]
		Echinaster sp.	X		Whole animal	[18,40]
	Valvatida	*Oreaster reticulatus*	X		Whole animal	[38,75,29,21,49,18,40,31]
**Mollusca**	Architaenioglossa**	*Pomacea lineata*	X			[49]
	Littorinimorpha	*Cassis tuberosa*	X			[21]
		Littorina angulifera	X		Flesh	[21,49]
		Strombus pugilis	X			[21]
	Lucinida	*Phacoides pectinatus*	X			[21]
	Myida	*Neoteredo reynei*	X			[21]
		Teredo pedicellata	X			[21]
	Myopsida	*Loligo sp.*	X		Shell	[49]
	Mytilida	*Mytella charruana*	X	X		[21,10]

		Mytella guyanensis	X	X	Flesh, shell	[21,10,49,79]
	Neogastropoda	*Pugilina morio*	X			[21]
	Ostreida	*Crassostrea rhizophorae*	X	X	Shell	[21,10,49,79]
	Stylommatophora	"slug"	X		Whole animal	[18]
		Megalobulimus oblongus	X		Whole animal	[38]
	Venerida	*Anomalocardia brasiliana*	X	X		[21,10,49]
		Lucina pectinata	X			[21]
**Cnidaria**	Siphonophorae	*Physalia physalis*	X		Whole animal	[21,49,79]
	Scleractinia	*Mussismilia harttii*	X			[21]

** Superfamily
