# Supplementary material for: Wildlife as Food and Medicine in Brazil: A Neglected Zoonotic Risk?
Source: Pathogens. 2024 Mar 2;13(3):222. doi: 10.3390/pathogens13030222 (PMC10975579; doi:10.3390/pathogens13030222)
Supplement: Supplementary file 1 [file pathogens-13-00222-s001.zip › Table S2.pdf]

Table S2: Sources of ethnobiological data explored in the manuscript.

Reference #	DOI	Title	Animals as Medicine	Food	Obs
33	10.1007/s10661-011-2465-0	A review on human attitudes towards reptiles in Brazil	X	X	Focus on the exploitation and relationship with the taxon. Includes other uses
25	10.1186/1746-4269-8-27	A zoological catalogue of hunted reptiles in the semiarid region of Brazil	X	X	Focus on the exploitation of the taxon. Includes other uses
31	10.1186/1472-6882-9-17	Animal-based folk remedies sold in public markets in Crato and Juazeiro do Norte, Ceará, Brazil	X		
37	10.1590/s0001-37652012005000038	Animal-based medicines used in ethnoveterinary practices in the semi-arid region of Northeastern Brazil	X		
	10.1186/1472-6882-8-44	Animal-based remedies as complementary medicines in Santa Cruz do Capibaribe, Brazil	X		
38	10.1093/ecam/nep134	Animal-based remedies as complementary medicines in the semi-arid region of northeastern Brazil	X		
20	10.1007/s10745-012-9516-1	Animals for the Gods: Magical and Religious Faunal Use and Trade in Brazil			Religious and/or magical only
30	10.1186/1746-4269-5-37	Animals to heal animals: ethnoveterinary practices in semiarid region, northeastern Brazil	X		
	10.1186/1746-4269-9-3	Birds and people in semiarid northeastern Brazil: symbolic and medicinal relationships	X		
43	10.1186/1746-4269-10-8	Chain of commercialization of Podocnemis spp. turtles (Testudines: Podocnemididae) in the Purus River, Amazon basin, Brazil: current status and perspectives		X	
29	10.1016/j.jep.2009.04.049	Commercialization of animal-derived remedies as complementary medicine in the semi-arid region of Northeastern Brazil	X		

32	10.1186/1746-4269-6-29	Ethnopharmacological survey among migrants living in the Southeast Atlantic Forest of Diadema, São Paulo, Brazil	X		
	10.1186/1746-4269-5-1	Fauna used in popular medicine in Northeast Brazil	X		
49	10.1016/j.jep.2006.03.007	From cnidarians to mammals: the use of animals as remedies in fishing communities in NE Brazil	X		
11	10.15451/ec2016-7-5.5-1-51	Game mammals of the Caatinga biome	X	X	Focus on general exploitation of the taxon. Other non-ingestion uses included
	10.1186/s13002-017-0174-7	Habits and customs of crab catchers in southern Bahia, Brazil		X	Focus on methods of sourcing
47	10.1186/1746-4269-5-36	Hunting and use of terrestrial fauna used by Caiçaras from the Atlantic Forest coast (Brazil)	X	X	
46	10.1186/1746-4269-5-12	Hunting strategies used in the semi-arid region of northeastern Brazil			Focus on methods of sourcing
27	10.1155/2012/474716	Ichthyofauna used in traditional medicine in Brazil	X		
39	10.1186/s13002-020-00365-5	Illegal trade of songbirds: an analysis of the activity in an area of northeast Brazil			Rearing only
50	10.1016/j.jnc.2019.02.002	Keeping reptiles as pets in Brazil: Ethnozoological and conservation aspects			Rearing only
	10.3855/jidc.10177	Knowledge, practice and perception of human-marsupial interactions in health promotion	X	X	Focus on the general exploitation of the taxon
22	10.1186/1746-4269-10-81	Local knowledge and exploitation of the avian fauna by a rural community in the semi-arid zone of northeastern Brazil	X	X	includes pet rearing
12	10.1186/s13002-018-0276-x	Local knowledge, use, and conservation of wild birds in the semi-arid region of Paraíba state, northeastern Brazil	X	X	Focus on the exploitation of the taxon. Includes rearing
23	10.1016/j.biocon.2017.06.013	Market access and wild meat consumption in the central Amazon, Brazil		X	

40	10.1159/000235855	Medicinal animals as therapeutic alternative in a semi-arid region of northeastern Brazil	X	
24	10.1186/1746-4269-7-30	Medicinal animals used in ethnoveterinary practices of the 'Cariri Paraibano', NE Brazil.	X	
26	10.1186/1746-4269-8-37	Medicinal use of fauna by a traditional community in the Brazilian Amazonia	X	
	10.1186/1472-6882-9-35	Termite usage associated with antibiotic therapy: enhancement of aminoglycoside antibiotic activity by natural products of Nasutitermes corniger (Motschulsky 1855)	X	
21	10.1641/B571107	The Role of Animal-derived Remedies as Complementary Medicines in Brazil	X	
	10.1186/1746-4269-3-32	The use of zootherapeutics in folk veterinary medicine in the district of Cubati, Paraíba State, Brazil	X	
19	10.1007/s10745-010-9352-0	Trade of animals used in Brazilian traditional medicine: trends and implications for conservation.	X	
36	10.1186/1746-4269-8-41	Traditional uses of medicinal animals in the semi-arid region of northeastern Brazil	X	
10	10.1155/2013/670352	Wild animals used as food medicine in Brazil	X	X
35	10.1007/s10745-019-00107-6	Wild Meat Trade and Consumption in the Central Amazon, Brazil		X
	10.1016/j.jep.2006.10.033	Zootherapeutic practices among fishing communities in North and Northeast Brazil: a comparison	X	
13	10.1186/s13002-018-0259-y	Zootherapeutic uses of wildmeat and associated products in the semiarid region of Brazil: general aspects and challenges for conservation.	X	
41	10.1186/1746-4269-5-21	Zootherapeutics utilized by residents of the community Poço Dantas, Crato-CE, Brazil	X	
18	10.1016/j.jep.2007.07.015	Zootherapy goes to town: the use of animal-based remedies in urban areas of NE and N Brazil	X	
